# Case report: A rare Salter–Harris V metaphyseal fatigue fracture of the knee in an adolescent patient with obesity

**DOI:** 10.3389/fped.2023.1209369

**Published:** 2023-06-22

**Authors:** Chao Gao, Ling Le Feng, Jiang Hua Zheng, Jin Cao, Hua Jing Sun

**Affiliations:** ^1^ Department of Orthopedics, Ningbo Sixth Hospital, Ningbo, China; ^2^ Department of Clinical Laboratory, Wu Xiang Health Center, Ningbo University Affiliated People's Hospital, Ningbo, China

**Keywords:** fatigue fracture, Salter V fracture, obese adolescent, metaphysis, operative treatment

## Abstract

Stress fractures are rare, occurring in 1.5/100,000 high school athletes. High impact, repetitive loading participation in woman's sports, and being a white athlete have been identified as risk factors for stress fractures. Mostly treated conservatively, they are more common in the tibia (33%). Stress fractures requiring surgery, which are extremely rare, have been reported in the scaphoid, fifth metatarsal, and neck of femur. Herein, a 16-year-old adolescent patient with obesity presented with atypical knee pain after prolonged exercise. Advanced imaging revealed a stress fracture of the left tibia with a Salter–Harris type V fracture and varus deformity of the knee. We initially managed the fatigue fracture conservatively, followed by surgical correction of the varus deformity in the knee joint. The patient made a satisfactory recovery with equal limb length and no evidence of claudication. This is the first case of a proximal tibial metaphyseal stress fracture requiring surgery. The clinical manifestations of proximal tibial metaphyseal stress fractures and potential treatment strategies and the use of magnetic resonance for tibial stress fractures have been discussed. Understanding the location of unusual stress fractures can improve early diagnostic efficiency and reduce complication rates, healthcare costs, and recovery time.

## Introduction

Bone formation and breakdown is delicately balanced under the regulatory control of the skeletal endocrine system. However, repetitive loading (1) that exceeds the skeletal auto-repair capacity may lead to stress fractures, an overuse injury commonly observed in athletes (2, 3). Approximately 95% of such fractures have been reported in the lower extremities, most commonly in the proximal tibia (4), and most fatigue fractures are treated conservatively. Herein, a 16-year-old boy was diagnosed with a rare fatigue fracture of the tibial metaphysis combined with a Salter–Harris V fracture who developed a varus deformity caused by prolonged absence and eventually required surgery. Although fatigue fractures are widely reported, the present patient's severe disease is unique, which provides an opportunity for a more comprehensive understanding of such diseases so that limiting their management to conservative treatment can be avoided.

## Case report

A 16-year-old boy visited the orthopedic clinic because of pain and stiffness in his left knee. He denied any history of trauma. The patient was obese (body mass index: 40 kg/m^2^) and had been exercising 4 h/day to reduce weight for a long time. He started having intermittent pain in his left knee joint 3 years after initiating this exercise regimen. Because the pain dissipated at rest, he did not initially seek treatment. His pain significantly worsened after 1 year of continued exercise and did not completely dissipate at rest. The patient reported no significant medical history other than obesity.

**Figure 1:**
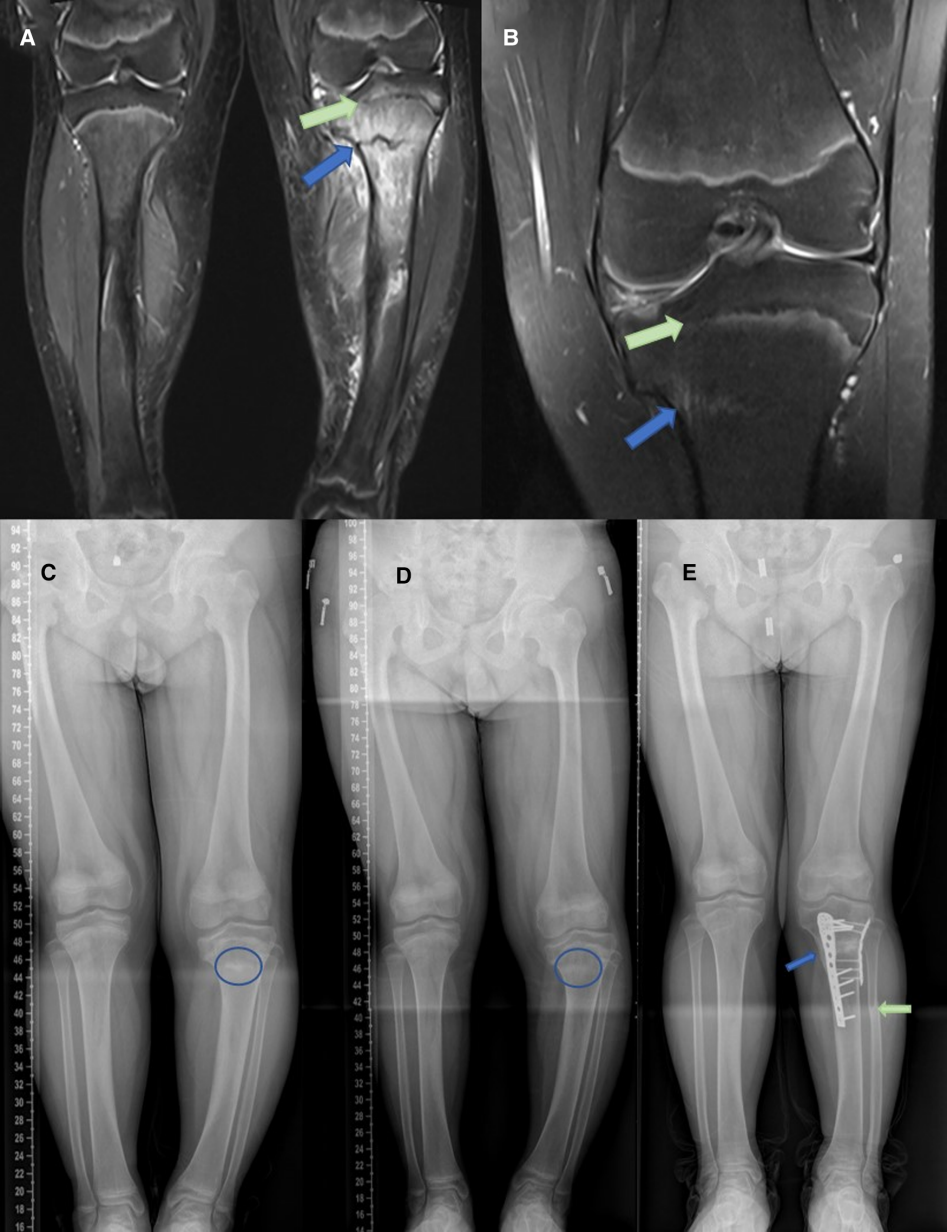
(**A**) T2-weighted MRI demonstrated a rough, high signal fracture line, surrounded by extensive high signal bone marrow edema (blue arrow) with a compression injury to the medial tibial growth plate (Salter V fracture) (green arrow). (**B**) After 3 months of conservative treatment, the stress fracture line disappeared and the Salter V fracture was clearly observed (**C–E**). Changes of the patient's lower limb force line. The blue circle represents the range of stress fractures. The arrow represents the location of the osteotomy.

On physical examination, the medial proximal tibia was tender without any significant swelling. Although there was no significant pain at rest, the patient experienced pain on deep palpation or weight-bearing. Moreover, although the range of motion was 0°–130° in both knees, the left knee was painful during movement. Ligaments and menisci were normal. Radiographs of the left knee showed varus deformity (Figure 1C), and magnetic resonance imaging (MRI) revealed a Salter–Harris V fatigue fracture at the proximal tibial metaphysis (Figure 1A).

**Figure 2:**
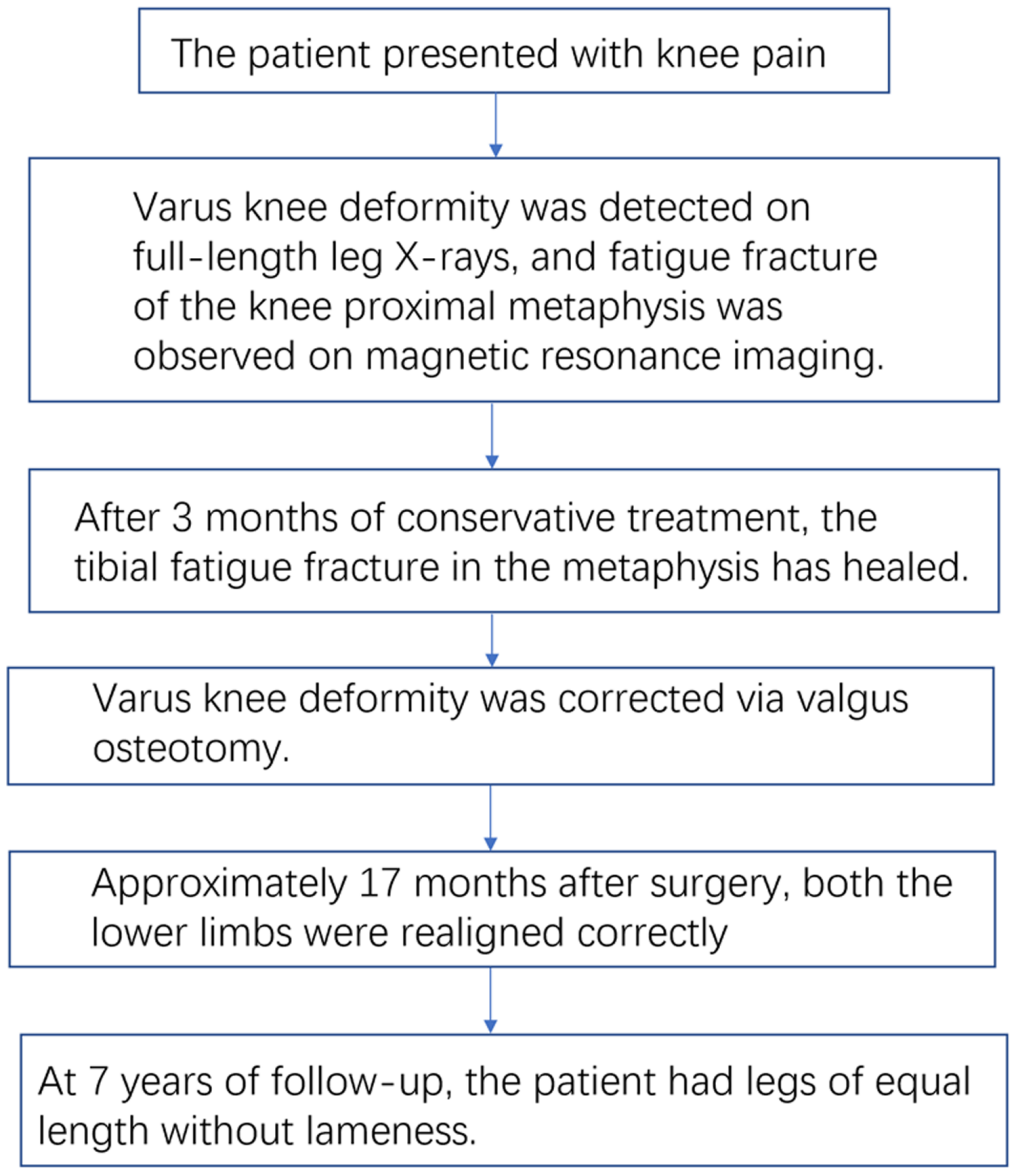
Timeline of patient treatment.

With conservative treatment, the stress fracture healed after 3 months, and valgus osteotomy of the knee was performed (Figure 1B). In brief, fibula osteotomy was performed under sterile conditions by making an incision from the upper to the middle lateral third of the lower leg. Then, an anteromedial incision was made on the proximal tibia. Valgus osteotomy was performed to correct the varus deformity and tibial internal rotation deformity, following which the lower limb could be realigned correctly. Finally, the osteotomy site was filled with allogeneic bone and fixed using two compression plates. The patient recovered 17 months after surgery, and the force line of both legs was significantly improved compared with the preoperative condition (Figure 1E). At a recent telephonic follow-up, the patient reported good recovery with both legs roughly equal in length and without claudication after 7 years (Figure 2).

## Discussion

Stress fractures result from the repetitive accumulation of microtrauma to the bone over time and are common in overuse injuries in military cadets and in actively training athletes (5). Etiologically, stress fractures can be classified into two categories: fatigue and insufficiency fractures. The former is caused by abnormal stress on normal bone, thereby disrupting the balance of bone formation and decomposition. By contrast, the latter is caused by normal stress acting on abnormal bone. In juvenile patients, as the metaphysis is a new cancellous bone, it is structurally fragile (6). The metaphysis is more fragile during the second peak of development, increasing the risk of injury. Stress fractures in teenagers include the characteristics of fatigue fractures and insufficiency fractures, which are special fractures.

Diagnosing stress fracture is difficult when the symptoms are atypical. The principal symptom of a stress fracture is localized pain that worsens with activity and decreases with rest. In this patient, physical examination revealed local sensitivity and tenderness with swelling. However, this often occurs in various other conditions. Studies have reported the need to examine the range of motion and perform provocation tests to reveal pain in deep stress fractures. In the vertical single leg hop test (7), a provocation test for diagnosing a stress fracture of the lower extremity, the patient is asked to jump on one leg; the test is positive when the patient has intense local pain.

According to current reports, fatigue fractures requiring surgical intervention frequently manifest at the femoral neck (8), anterior tibia (9), and fifth metatarsal bone (10). The majority of metaphyseal tibial fatigue fractures are managed conservatively. Li et al. (11) reported the case of a 13-year-old female adolescent with no history of trauma. MRI revealed a proximal tibial metaphyseal stress fracture, and the patient recovered well after conservative treatment. Ali et al. (12) reported the case of a 23-year-old woman, also without any history of trauma, who developed painful discomfort in both knees after a month of intense exercise in preparation for a police examination. MRI in this patient revealed bilateral metaphyseal stress fractures of the proximal tibias. Her vitamin D levels were only 8 ng/ml (normal: 25–100 ng/ml). The patient recovered poorly after rest and vitamin D supplementation. However, following transcutaneous electrical stimulation, ultrasound, hot packs, and physical exercise, the patient experienced gradually decreasing levels of pain.

Of note, the positive manifestations of stress fractures such as parallel periosteal reaction and signs of callus formation do not appear until 3–4 weeks after symptom onset (13, 14). Therefore, early x-ray examination is usually difficult and poorly sensitive; x-ray may sometimes be negative. MRI is the gold standard for diagnosing fatigue fractures. A basic T1-weighted MRI sequence can provide specific anatomical details and better display the bone structure. T1-weighted, T2-weighted fat-suppression sequence, and short tau inversion recovery sequence images are particularly important for judging the degree of bone marrow edema and soft-tissue injury (15).

We acknowledge a limitation of this study. Corrective surgery was performed on the 16-year-old patient before reaching adulthood. Although there is a risk of deformity recurrence, prolonged uncorrected varus deformity can result in joint degeneration. Upon careful examination of preoperative radiographs, it was observed that the patient had developed wear on the medial condyle of their knee. Therefore, additional correction is necessary. Moreover, as the patient was 16 years old and had already passed their peak growth and development stage with low potential for further growth, valgus osteotomy can be deemed acceptable.

## Conclusions

This report analyzed the possibility that the relative structural fragility of the metaphysis and increased physical activity during adolescence may increase the risk of lower extremity stress fractures in adolescents. Moreover, the importance of imaging during diagnosis and rehabilitation and the need for the continuous monitoring of patients returning to exercise after a stress fracture were discussed. Learning about unusual stress fractures will help clinicians diagnose it quickly to avoid delayed diagnosis or misdiagnosis, prevent complications, and promote early recovery.

## Data Availability

The original contributions presented in the study are included in the article, further inquiries can be directed to the corresponding author.
